# Neuroimmunological overlap syndromes in optic neuritis, myelitis, and connective tissue diseases

**DOI:** 10.3389/fneur.2026.1887536

**Published:** 2026-07-22

**Authors:** Shu-ting Yang, Zhi-wen Wang

**Affiliations:** 1 Department of Neurology, Huizhou Central People’s Hospital, Huizhou, China; 2 Department of Rheumatology and Immunology, Huizhou Central People’s Hospital, Huizhou, China

**Keywords:** biomarker-based diagnosis, neuroimmune crosstalk, neuroimmunological overlap syndromes, pathogenic mechanisms, targeted immunotherapy

## Abstract

**Background:**

Neuroimmunological overlap syndromes encompassing optic neuritis, myelitis, and connective tissue disorders (CTDs) represent an emerging autoimmune spectrum at the intersection of neurology, immunology, and rheumatology. These conditions share convergent pathogenic mechanisms including blood–brain barrier dysfunction, cytokine dysregulation, complement activation, and adaptive immune imbalance, yet are often managed as discrete entities despite overlapping clinical features.

**Method:**

A narrative review was conducted across PubMed, Web of Science, and Google Scholar (January 2014–March 2026) using keywords related to NMOSD, MOGAD, MS, optic neuritis, myelitis, and CTDs. Preference was given to systematic reviews, meta-analyses, randomized controlled trials, and large observational cohort studies.

**Outcomes:**

Shared pathogenic mechanisms include BBB disruption, pro-inflammatory cytokines (IL-6, IL-17, TNF-*α*, IFN-*γ*), complement activation, and B-cell/Th17-mediated autoimmunity. Disease-defining antibodies (AQP4-IgG, MOG-IgG) have refined diagnostic accuracy. Clinically, optic neuritis and myelitis serve as principal overlapping manifestations complicating differentiation among NMOSD, MOGAD, MS, and CTD-associated neuroinflammation. Four biologics inebilizumab (B-cell depletion), satralizumab (IL-6 blockade), and eculizumab/ravulizumab (complement inhibition) are approved for AQP4-IgG-positive NMOSD. However, no targeted therapies exist for MOGAD, and CTD-associated neuroinflammation lacks trial data. Emerging biomarkers (GFAP, NfL) and precision medicine tools remain unvalidated for routine clinical use.

**Conclusion:**

Neuroimmunological overlap syndromes should be conceptualized as a unified neuroimmune continuum rather than discrete diseases. Priority research includes biomarker validation, head-to-head comparative cohorts, and randomized trials for MOGAD and CTD-associated neuroinflammation.

## Graphical abstract

**Figure:**
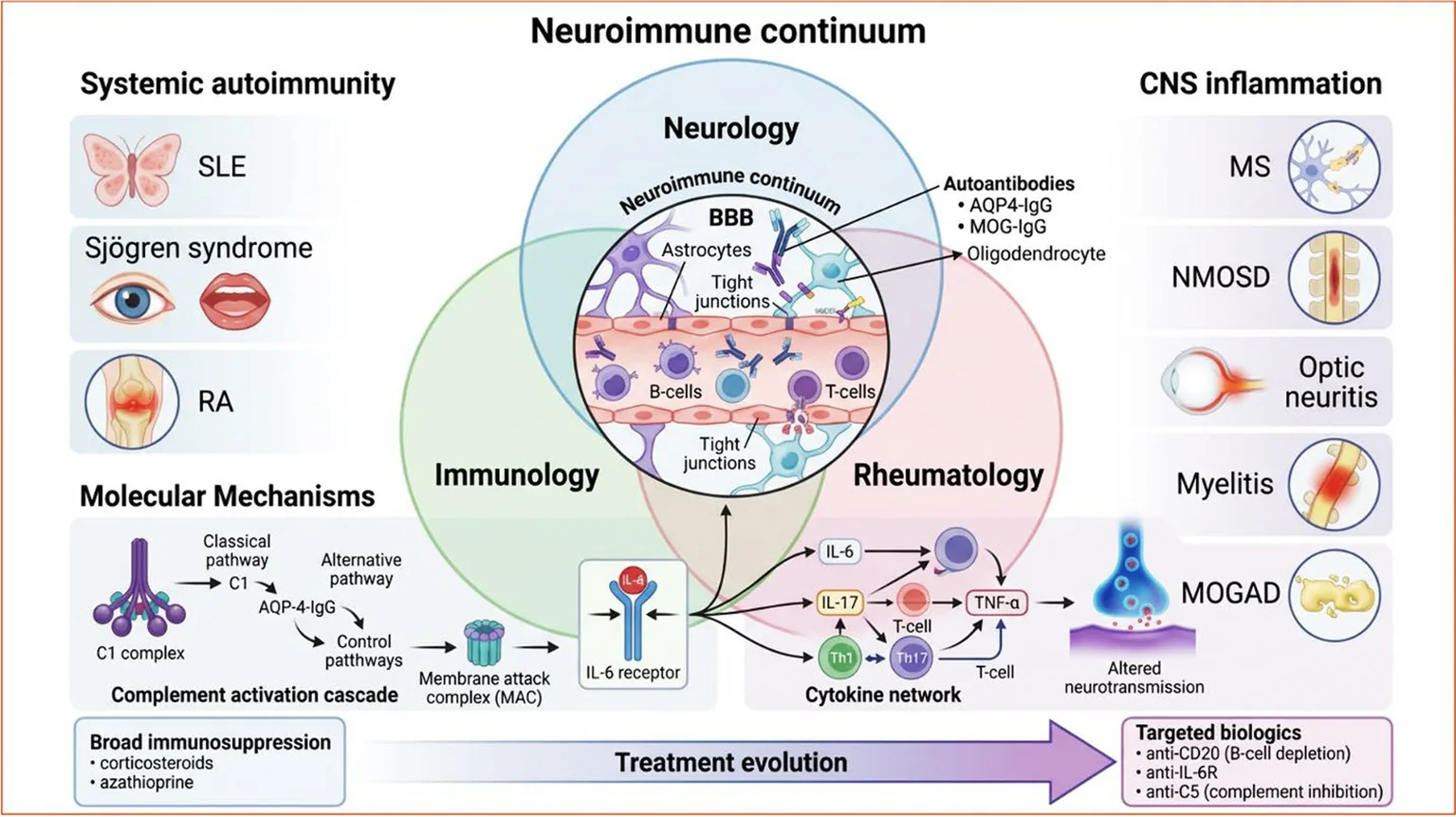
Graphical abstract

## 1 Introduction

Neuroimmunological overlap syndromes represent a group of conditions in which autoimmune-mediated central nervous system (CNS) inflammation co-occurs with systemic connective tissue disorders (CTDs). The nervous and immune systems exhibit bidirectional interactions through the peripheral nervous system (PNS), forming neuroimmune cell units that regulate inflammation and maintain tissue homeostasis (1, 2). Various cytokines, inflammatory mediators, and neurotransmitters produced by nerve and immune cells create feedback loops between immune cells and central, peripheral, sympathetic, and parasympathetic nerves (3). The blood–brain barrier (BBB) normally protects the CNS from circulatory inflammatory signals, but injury to the CNS disrupts this balance through interference with the sympathetic nervous system, PNS, and hypothalamic–pituitary–adrenal axis (4, 5).

The focus on optic neuritis, myelitis, and CTDs is driven by their clinical mimicry, high morbidity when misdiagnosed, and shared immunopathology. Psychiatric comorbidities including depression, anxiety, and mood disturbances are common in RA patients, adversely affecting quality of life, treatment adherence, and biological therapy response (6–8). Neuromyelitis optica spectrum disorder (NMOSD) exemplifies this overlap; traditionally considered a monophasic disorder with bilateral optic neuritis and transverse myelitis, it now includes relapsing cases with aquaporin-4 antibodies (AQP4-IgG) and coexisting autoimmune disorders such as SLE or Sjögren syndrome (9, 10). Chronic peripheral inflammation in RA is closely linked to CNS involvement including meningitis, rheumatoid nodules, and cerebral vasculitis (11, 12). Some MS immunotherapies may aggravate NMO, underscoring the need for early and accurate diagnosis (13, 14).

Several existing reviews address NMOSD, MOGAD, or CTD-associated neurological disease individually, but few examine their shared pathophysiology, overlapping biomarker landscape, and divergent treatment pathways within a unified framework. The novel contribution of this review is threefold: (i) it integrates molecular and cellular pathways linking systemic autoimmunity (SLE, SS, RA) to CNS injury with disease-specific biology of NMOSD, MOGAD, and MS within a neuroimmune-continuum framework; (ii) it contrasts treatment pathways for AQP4-IgG-seropositive NMOSD supported by four approved biologics with distinct mechanisms against the under-validated treatment landscape for MOGAD and CTD-associated neuroinflammation; and (iii) it critically appraises emerging biomarkers (GFAP, NfL, single-cell transcriptomics) and precision-medicine tools, distinguishing validated utility from promising but unproven research directions.

The central organizing concept is the “neuroimmune continuum”-a framework positing that neuroimmunological overlap syndromes are interconnected manifestations of shared pathogenic mechanisms rather than discrete disease entities. This approach challenges categorical diagnosis and treatment, proposing stratification along a continuum based on dominant pathogenic pathways (astrocytopathy, demyelination, microglial activation, or vasculopathy). The framework explains the frequent co-occurrence of CNS inflammation and systemic autoimmunity, accounts for overlapping clinical features, and guides therapeutic selection based on underlying immunopathology. Throughout this review, we apply this continuum framework to synthesize evidence across traditional disease boundaries, identify shared molecular targets, and highlight areas where the continuum model generates testable predictions for future research.

## 2 Methodology

### 2.1 Search strategy

This narrative review was developed through a systematic literature search of PubMed, Web of Science Core Collection, and Google Scholar. The search was conducted between January 2014 and March 2026 to identify English-language articles published with additional historical references included where they provided essential foundational context. The search strategy combined Medical Subject Headings (Bhol et al.) terms and free-text keywords using the following Boolean operators: (“neuromyelitis optica spectrum disorder” OR “NMOSD” OR “MOG antibody-associated disease” OR “MOGAD” OR “optic neuritis” OR “myelitis”) AND (“connective tissue disease” OR “systemic lupus erythematosus” OR “Sjögren syndrome” OR “rheumatoid arthritis” OR “neuroimmune crosstalk” OR “blood–brain barrier”). Additional targeted searches were performed for specific topics including “AQP4-IgG,” “MOG-IgG,” “GFAP biomarker,” “neurofilament light chain,” “B-cell depletion,” “complement inhibition,” and “IL-6 receptor blockade.” To quantify the literature landscape, bibliometric analyses were consulted, including a study of 3,027 papers on optic neuritis published and 2,576 papers on MOGAD, and the rapidly growing body of NMOSD treatment literature.

### 2.2 Inclusion and exclusion criteria

Articles were included if they: (1) addressed the pathophysiology, diagnosis, or treatment of NMOSD, MOGAD, multiple sclerosis, or connective tissue disease-associated neurological involvement; (2) focused on optic neuritis, myelitis, or related CNS inflammatory syndromes; (3) were published in peer-reviewed journals; and (4) were written in English. Articles were excluded if they: (1) were case reports with fewer than three patients unless they described novel or rare phenomena; (2) were conference abstracts without full-text availability; (3) focused exclusively on peripheral nervous system involvement without CNS correlation; or (4) were duplicate publications. Preference was given to systematic reviews, meta-analyses, randomized controlled trials, and large observational cohort studies. For emerging topics where such evidence was limited, expert consensus statements and high-quality narrative reviews were also included.

### 2.3 Study selection and data extraction

Two authors (S.T.Y. and Z.W.W.) independently screened titles and abstracts for eligibility. Full texts of potentially relevant articles were retrieved and assessed against the inclusion criteria. Disagreements were resolved through discussion and consensus. Data were extracted using a standardized form that captured: (1) study characteristics (author, year, design, sample size); (2) disease category (NMOSD, MOGAD, MS, CTD); (3) key pathogenic mechanisms described; (4) diagnostic biomarkers reported; (5) therapeutic interventions and evidence level; and (6) main findings and conclusions.

### 2.4 Evidence synthesis

Given the heterogeneous nature of the included literature spanning basic science, translational research, clinical studies, and therapeutic trials-a narrative synthesis approach was adopted. Findings were organized thematically around the “neuroimmune continuum” conceptual framework (see Section 2.5), with evidence synthesized across traditional disease boundaries to identify shared pathogenic mechanisms, overlapping biomarker profiles, and divergent treatment responses. The strength of evidence for therapeutic recommendations was graded according to study design, with randomized controlled trial data considered highest quality, followed by prospective cohort studies, retrospective analyses, and expert consensus. Throughout the review, we explicitly distinguish between established clinical biomarkers (AQP4-IgG, MOG-IgG, CSF oligoclonal bands), promising but unvalidated candidates (GFAP, NfL), and experimental research tools (single-cell transcriptomics, proteomic signatures, AI-based models).

## 3 Pathophysiology and molecular mechanisms

Neuroimmunological overlap syndromes involving optic neuritis, myelitis, and connective tissue disorders (CTDs) arise from complex interactions between the immune system and the central nervous system (CNS). Increasing evidence suggests that autoimmune-mediated inflammation, disruption of the blood–brain barrier (BBB), cytokine dysregulation, and abnormal activation of immune cells collectively contribute to disease initiation and progression. These molecular and immunological abnormalities play critical roles in disorders such as neuromyelitis optica spectrum disorder (NMOSD), multiple sclerosis (MS), systemic lupus erythematosus (SLE), Sjögren syndrome (SS), and rheumatoid arthritis (RA).

### 3.1 Blood–brain barrier dysfunction

The BBB is a highly selective barrier composed of endothelial cells, astrocytes, and pericytes that protects the CNS from harmful circulating molecules and immune cells. Under normal physiological conditions, the BBB maintains CNS immune privilege and regulates molecular transport between the blood and neural tissues (15). However, inflammatory and autoimmune conditions can disrupt BBB integrity, leading to increased permeability and infiltration of autoreactive immune cells into the CNS.

In autoimmune neuroinflammatory disorders, cytokines such as tumor necrosis factor-alpha (TNF-*α*), interleukin-6 (IL-6), and interferon-gamma (IFN-*γ*) contribute to endothelial dysfunction and tight-junction disruption (16). BBB impairment allows activated T cells, B cells, macrophages, and pathogenic antibodies to enter the CNS, where they induce inflammation, demyelination, and neuronal injury (17).

In NMOSD, AQP4-IgG antibodies cross the damaged BBB and target aquaporin-4 water channels expressed on astrocytes, leading to complement-mediated astrocytic destruction and secondary demyelination (18). Similarly, in SLE and RA, systemic inflammatory mediators can alter BBB permeability and contribute to neuropsychiatric and demyelinating manifestations (19).

### 3.2 Cytokines and inflammatory pathways

Cytokines and chemokines play essential roles in neuroimmune communication and autoimmune inflammation. Elevated levels of pro-inflammatory cytokines including IL-1β, IL-6, IL-17, TNF-*α*, and IFN-*γ* have been identified in patients with autoimmune demyelinating diseases and CTDs. These cytokines regulate immune-cell activation, leukocyte migration, and tissue injury within the CNS (20).

Among these mediators, IL-6 is considered particularly important in NMOSD pathogenesis because it promotes B-cell survival, antibody production, BBB disruption, and differentiation of pathogenic T helper 17 (Th17) cells (21). Increased IL-17 production further amplifies inflammatory cascades and neutrophil recruitment, enhancing CNS tissue damage (22). TNF-α and IFN-γ also contribute to oligodendrocyte injury, neuronal dysfunction, and chronic neuroinflammation (23). Chemokines such as CXCL13 and CCL2 regulate immune-cell trafficking into inflamed neural tissues (24). In RA and SLE, persistent systemic inflammation may induce chronic activation of microglia and astrocytes, contributing to cognitive dysfunction, psychiatric symptoms, and neurodegeneration (25). Moreover, complement activation is another major inflammatory pathway involved in autoimmune demyelination, particularly in AQP4-IgG-positive NMOSD.

### 3.3 Autoantibodies

Autoantibodies are among the most important biomarkers and pathogenic mediators in neuroimmunological overlap syndromes. The discovery of aquaporin-4 immunoglobulin G (AQP4-IgG) revolutionized the understanding of NMOSD and enabled differentiation from MS (26). AQP4-IgG targets astrocytic water channels, causing complement-dependent cytotoxicity, astrocyte loss, and secondary demyelination (27).

Another important antibody is myelin oligodendrocyte glycoprotein immunoglobulin G (MOG-IgG), which is associated with MOG antibody-associated disease (MOGAD), a condition characterized by optic neuritis, myelitis, and acute disseminated encephalomyelitis-like presentations. Compared with AQP4-IgG-positive NMOSD, MOGAD demonstrates different radiological and clinical characteristics (28).

In CTDs, several systemic autoantibodies have been associated with neurological manifestations. Antinuclear antibodies (ANA), anti-double-stranded DNA (anti-dsDNA), anti-Ro/SSA, and anti-La/SSB antibodies are frequently detected in SLE and Sjögren syndrome patients with CNS involvement (29). These antibodies may contribute to vasculitis, endothelial dysfunction, and immune-mediated neuronal injury. In some patients, overlap between systemic autoimmunity and CNS demyelination suggests shared immunopathogenic mechanisms (30).

### 3.4 T-cell and B-cell dysregulation

**Figure 1:**
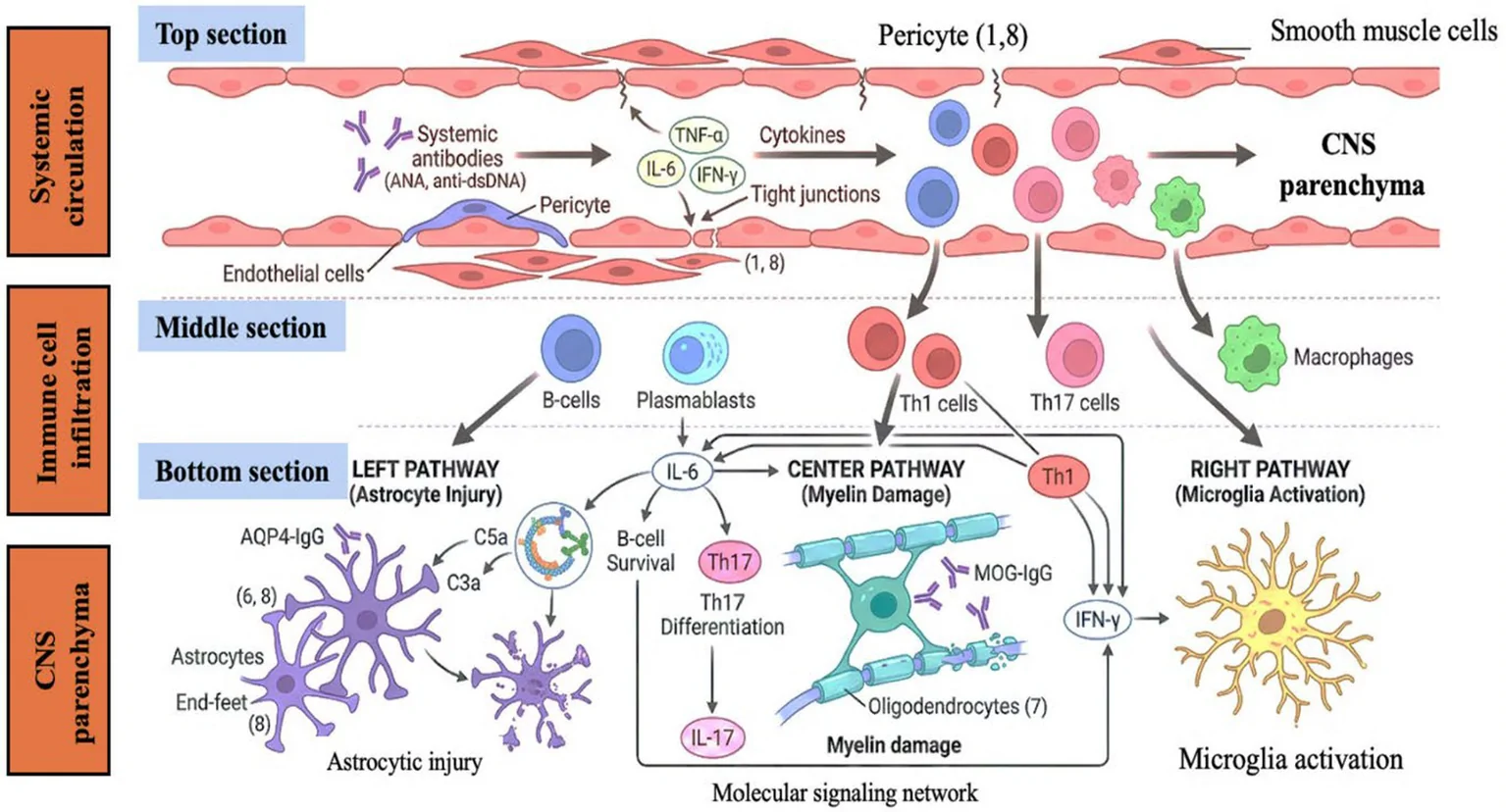
Pathophysiology of neuroimmunological overlap syndrome. Schematic representation of the molecular and cellular mechanisms underlying neuroimmunological overlap syndromes. Established pathways (AQP4-IgG-mediated complement activation, IL-6-driven Th17 differentiation) are shown in solid lines; hypothetical or emerging pathways (neuroimmune continuum integration, shared cytokine networks across disease boundaries) are shown in dashed lines. AQP4-IgG, aquaporin-4 immunoglobulin G; MOG-IgG, myelin oligodendrocyte glycoprotein immunoglobulin G; BBB, blood–brain barrier; Th1/Th17, T helper cell subsets.

Autoimmune neurological diseases arise from dysregulated adaptive immunity. T cells and B cells drive CNS inflammation and tissue injury through direct cytotoxicity, cytokine release, and autoantibody generation (31). CD4^+^ T helper cells, especially Th1 and Th17 subsets, are strongly implicated in autoimmune demyelinating disorders (32). Th17 cells produce IL-17 and stimulate neutrophil-mediated inflammation, while Th1 cells secrete IFN-*γ* and activate macrophages and microglia (33). Regulatory T cells (Tregs), which normally suppress excessive immune activation, are often functionally impaired in autoimmune diseases, leading to loss of immune tolerance. B cells also play multifaceted roles beyond antibody production. They function as antigen-presenting cells, secrete inflammatory cytokines, and promote T-cell activation. In NMOSD, plasmablast-derived AQP4-IgG production is a hallmark feature of disease activity. This understanding has led to the development of B-cell-targeted therapies such as rituximab, anti-CD20 monoclonal antibody and inebilizumab, an anti-CD19 monoclonal antibody that depletes a broader range of B-lineage cells, including plasmablasts and some plasma cells (34, 35). The molecular and cellular mechanisms underlying neuroimmunological overlap syndromes, including blood–brain barrier dysfunction, cytokine dysregulation, autoantibody-mediated injury, and T-cell/B-cell dysregulation, are schematically represented in Figure 1.

### 3.5 Neuroimmune continuum: a unified conceptual framework

The concept of a “neuroimmune continuum” represents a paradigm shift in understanding neuroimmunological overlap syndromes. Rather than viewing NMOSD, MOGAD, MS, SLE, SS, and RA as discrete categorical entities, the continuum framework posits that these conditions share overlapping pathogenic mechanisms and exist along a spectrum of neuroimmune dysregulation. This conceptualization is grounded in several key observations.

First, the molecular pathways underlying these conditions converge at multiple levels. BBB dysfunction, cytokine dysregulation (particularly IL-6, IL-17, TNF-*α*, and IFN-*γ*), complement activation, and adaptive immune imbalance characterized by B-cell hyperactivity and Th1/Th17 polarization are consistently identified across NMOSD, MS, SLE, SS, and RA. This shared inflammatory backbone explains why patients with one condition often develop features of another, such as NMOSD occurring in patients with SLE or SS, and why treatments targeting shared pathways (e.g., B-cell depletion) show efficacy across multiple conditions (34, 36).

Second, the continuum framework accommodates the heterogeneity observed within traditional diagnostic categories. For example, NMOSD patients may present with pure optic neuritis, pure myelitis, or combined syndromes; they may be AQP4-IgG-positive or seronegative; and they may or may not have coexisting CTDs. These variations do not represent distinct diseases but rather different positions along the neuroimmune continuum, determined by the dominant pathogenic mechanism, genetic susceptibility, and environmental triggers in each individual patient (37).

Third, the continuum framework has practical implications for diagnosis and treatment. It suggests that diagnostic evaluation should not be limited to identifying a single categorical diagnosis but should comprehensively assess the spectrum of possible pathogenic mechanisms present in each patient. Similarly, treatment selection should be guided by the dominant pathogenic pathway rather than by disease label alone. For instance, a patient with SLE-associated myelitis who is AQP4-IgG-positive may benefit from the same B-cell depletion or complement inhibition strategies used in classical NMOSD, even though their primary diagnosis is SLE (38, 39).

Fourth, the continuum framework generates testable predictions for future research. It predicts that (i) molecular signatures (transcriptomic, proteomic, epigenetic) will reveal continuous variation across diagnostic categories rather than discrete clustering; (ii) patients with different categorical diagnoses but similar dominant pathogenic pathways will respond similarly to targeted therapies; and (iii) longitudinal studies will show that patients can transition between phenotypic presentations over time as the balance of pathogenic mechanisms shifts. Testing these predictions requires prospective multi-omics studies with longitudinal follow-up and standardized treatment protocols-a priority area for future investigation (40, 41).

It is important to acknowledge that the continuum framework remains a hypothesis rather than an established fact. The degree to which shared molecular pathways reflect truly convergent mechanisms versus superficially similar downstream consequences of distinct upstream pathologies is incompletely understood. Head-to-head comparative cohorts across NMOSD, MOGAD, MS, and CTD-associated neuroinflammation are largely lacking, and the extent to which molecular signatures overlap or diverge across these conditions remains an open question. The continuum framework should therefore be understood as a heuristic for organizing current evidence and generating testable predictions, not as a validated diagnostic or classification system. Nevertheless, the continuum framework provides a useful heuristic for synthesizing current evidence, identifying knowledge gaps, and designing future studies that can rigorously test its validity.

Throughout this review, we apply the continuum framework to organize evidence, identify shared therapeutic targets, and highlight areas where the model generates actionable insights for clinical practice and research. The limitations and unresolved questions surrounding this framework are discussed in detail in Section 8.4.

## 4 Optic neuritis and myelitis in autoimmune diseases

Optic neuritis and myelitis are major inflammatory disorders of the central nervous system (CNS) commonly associated with autoimmune and demyelinating diseases. These conditions result from immune-mediated injury to the optic nerves and spinal cord, leading to visual impairment, sensory disturbances, motor dysfunction, and varying degrees of neurological disability. Although traditionally linked to multiple sclerosis (MS), optic neuritis and myelitis are increasingly recognized in neuromyelitis optica spectrum disorder (NMOSD), connective tissue diseases (CTDs), and other systemic autoimmune disorders (42). Accurate differentiation among these conditions is essential because their prognosis, pathophysiology, and therapeutic approaches differ substantially.

### 4.1 Neuromyelitis optica spectrum disorder (NMOSD)

NMOSD is a severe autoimmune astrocytopathic disease characterized primarily by recurrent episodes of optic neuritis and longitudinally extensive transverse myelitis (LETM) (38). The identification of aquaporin-4 immunoglobulin G (AQP4-IgG) antibodies has revolutionized the understanding and diagnosis of NMOSD. AQP4 is a water channel protein highly expressed on astrocytic foot processes within the CNS, particularly in the optic nerves, spinal cord, and periventricular regions. Binding of AQP4-IgG antibodies activates complement pathways, resulting in astrocyte injury, inflammatory demyelination, and neuronal damage (43).

Clinically, optic neuritis in NMOSD is bilateral, severe, and associated with poor visual recovery compared with MS-related optic neuritis (44). Patients may present with acute visual loss, pain during eye movement, impaired color vision, and visual field defects. Myelitis in NMOSD commonly manifests as LETM involving three or more contiguous vertebral segments, producing profound weakness, sensory loss, neuropathic pain, and bladder or bowel dysfunction (45). Some patients also develop area postrema syndrome characterized by persistent nausea, vomiting, and hiccups due to medullary involvement. MRI findings play a critical role in diagnosis. Spinal MRI typically demonstrates longitudinally extensive central cord lesions spanning multiple vertebral segments, often associated with cord swelling and edema (46).

Brain MRI may be normal in early disease or demonstrate lesions around the ventricles, hypothalamus, and brainstem where AQP4 expression is abundant. Optic nerve MRI frequently reveals extensive posterior optic nerve involvement and optic chiasm lesions. NMOSD frequently coexists with systemic autoimmune disorders such as systemic lupus erythematosus (SLE), Sjögren syndrome (SS), rheumatoid arthritis (RA), and autoimmune thyroid disease, supporting the concept of neuroimmunological overlap syndromes (39).

### 4.2 Myelin oligodendrocyte glycoprotein antibody-associated disease (MOGAD)

Myelin oligodendrocyte glycoprotein antibody-associated disease (MOGAD) has emerged as a distinct neuroimmunological entity characterized by serum antibodies targeting myelin oligodendrocyte glycoprotein (MOG-IgG), a protein expressed on the outermost surface of myelin sheaths and oligodendrocytes (47, 48). Unlike AQP4-IgG-positive NMOSD, which targets astrocytes, MOGAD primarily involves antibody-mediated and complement-dependent injury to myelin and oligodendrocytes, with relative preservation of astrocytes (28, 49). This fundamental difference in target cell population explains the distinct clinical, radiological, and therapeutic profiles of MOGAD compared to NMOSD.

Clinical Features: MOGAD presents with a spectrum of phenotypes including optic neuritis (the most common manifestation, affecting approximately 50–70% of patients), myelitis, acute disseminated encephalomyelitis (ADEM)-like presentations, and brainstem encephalitis (50, 51). Compared with AQP4-IgG-positive NMOSD, MOGAD-associated optic neuritis is more frequently bilateral, often involves the anterior optic nerve with optic disc edema and demonstrates better visual recovery following corticosteroid treatment (52, 53). Myelitis in MOGAD may present as longitudinally extensive transverse myelitis (LETM) resembling NMOSD, but conus medullaris involvement and brain abnormalities are more frequent in MOGAD (54, 55). Importantly, MOGAD more frequently follows a monophasic course (approximately 50–60% of patients), whereas NMOSD is typically relapsing (56).

Radiological Features: MRI findings in MOGAD differ from both NMOSD and MS. Spinal cord lesions in MOGAD are often longitudinally extensive (≥3 vertebral segments) with predilection for the conus medullaris and gray matter, whereas NMOSD lesions typically involve the central cord with swelling (57, 58). Brain MRI may be normal in up to 50% of MOGAD cases, but when abnormal, deep white matter, thalamic, and basal ganglia lesions are characteristic, distinguishing MOGAD from the periependymal lesions of NMOSD and the periventricular ‘Dawson’s fingers’ lesions of MS (59). Optic nerve MRI typically demonstrates bilateral, longitudinally extensive optic nerve involvement with predominant anterior segment enhancement in MOGAD, contrasting with the posterior, chiasmal involvement characteristic of NMOSD.

CSF and Serological Findings: CSF analysis in MOGAD typically shows lymphocytic pleocytosis and elevated protein, with oligoclonal bands being uncommon (≤20%) in contrast to MS (approximately 90%) (60, 61). MOG-IgG is detected by cell-based assays and may be transient, with titers declining during remission, unlike AQP4-IgG which is typically persistent (62). The transient nature of MOG-IgG has implications for diagnosis and monitoring, as seronegative conversion during remission is common and does not necessarily indicate disease resolution.

Disease Course and Prognosis: MOGAD has a more favorable prognosis than AQP4-IgG-positive NMOSD, with better recovery from acute attacks and lower relapse rates (60, 63). Approximately 50–60% of patients have a monophasic course, while relapsing disease occurs more frequently in adults than children. Long-term disability is generally milder than NMOSD but repeated optic neuritis attacks can lead to cumulative visual impairment. The Expanded Disability Status Scale (EDSS) scores at last follow-up are typically lower in MOGAD compared to NMOSD, reflecting the better overall prognosis.

Treatment: Acute attacks of MOGAD respond favorably to high-dose intravenous corticosteroids, with intravenous immunoglobulin (IVIG) or plasma exchange as second-line options (64, 65). Recent evidence supports the efficacy of IVIG in acute MOGAD attacks: Lotan et al. (66) conducted a retrospective multicenter study of 39 patients and demonstrated significant improvement in both EDSS and visual acuity measures following IVIG treatment (*p* < 0.0001 for both outcomes) (66). For patient’s refractory to corticosteroids, plasma exchange (PLEX) represents an important rescue therapy. Thakolwiboon et al. (67) reported on an international multicenter retrospective cohort of 234 MOGAD patients treated with PLEX, showing improvement in visual acuity from 20/400 to 20/20 (*p* < 0.001) and reduction in EDSS from a median of 4.0 to 1.0 (*p* < 0.001) (67). For relapse prevention, maintenance immunosuppression with azathioprine, mycophenolate mofetil, or rituximab is used, although rituximab appears less effective in MOGAD than in NMOSD (68, 69). Maintenance IVIG has also emerged as an effective strategy for relapse prevention. Chen et al. (70) conducted a retrospective cohort study of 59 adult patients with MOGAD and found that maintenance IVIG was associated with a significant reduction in annualized relapse rate from 1.4 pre-treatment to 0 while on therapy (70). Critically, the biologics approved for AQP4-IgG-positive NMOSD-eculizumab, ravulizumab, satralizumab, and inebilizumab-were specifically validated in seropositive cohorts and are not approved for MOGAD (71, 72). Two phase 3 clinical trials are currently evaluating the safety and efficacy of satralizumab (an IL-6 receptor inhibitor) and rozanolixizumab (an FcRn inhibitor) in relapsing MOGAD. Results of the METEOROID trial have been recently reported at the 2026 AAN annual meeting and are expected to be published in the near future. Corticosteroids and IVIG remain the mainstay of long-term management, reflecting the absence of MOGAD-specific pivotal trial data (65). Lotan et al. (73) provided a comprehensive review comparing immunotherapeutic strategies for NMOSD and MOGAD, highlighting the need for MOGAD-specific clinical trial data (73). This therapeutic gap represents a significant unmet need in the field.

**Figure 2:**
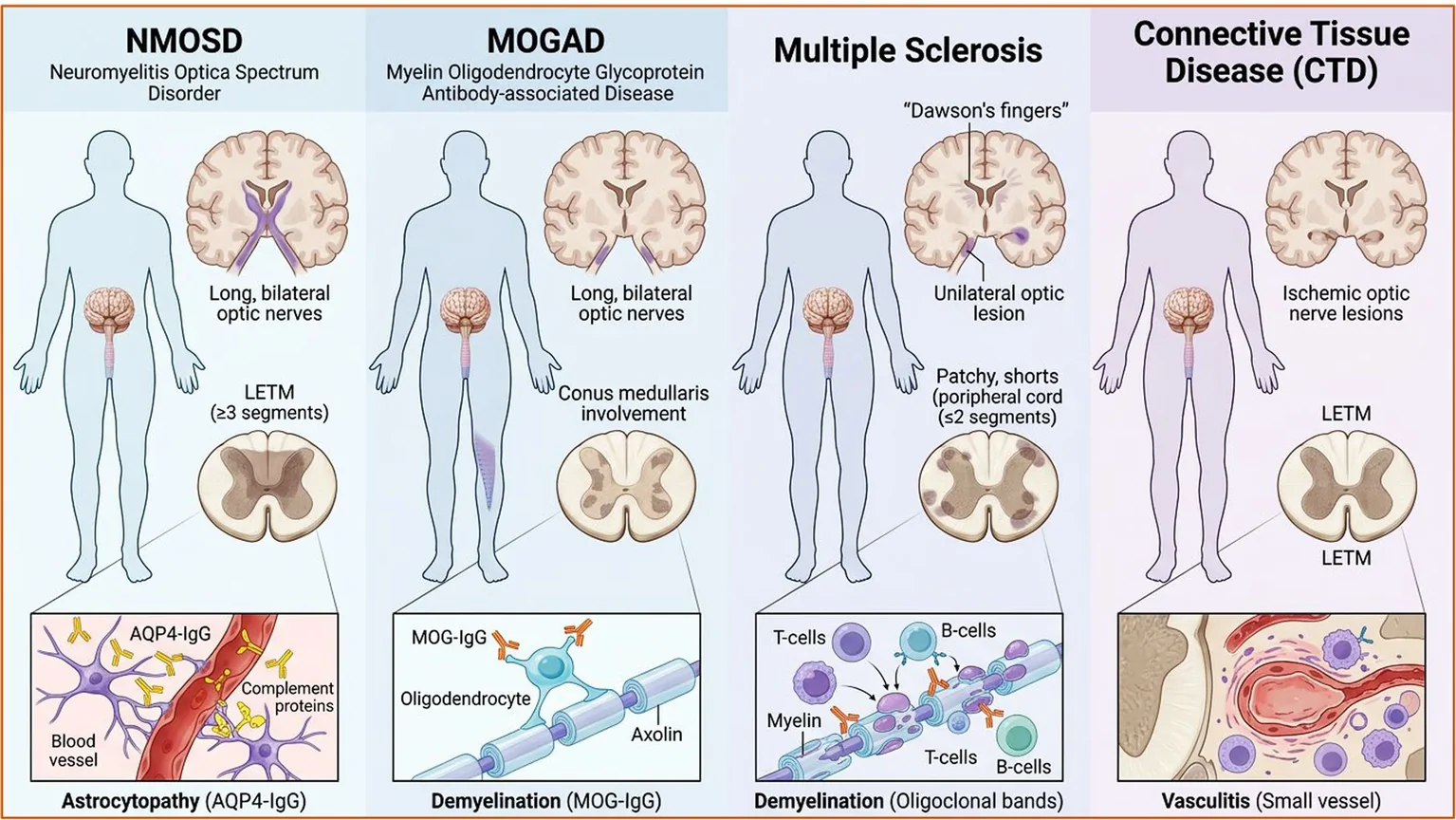
Comparative clinical and radiological features of optic neuritis and myelitis across neuroimmunological overlap syndromes. Quadrant comparison of four neuroimmunological conditions: NMOSD shows bilateral long-segment optic neuritis with posterior predilection and longitudinally extensive transverse myelitis (LETM, ≥3 segments) with central cord involvement, driven by AQP4-IgG-mediated astrocytopathy and complement activation. MOGAD depicts bilateral anterior optic neuritis and conus-involving LETM, mediated by MOG-IgG targeting oligodendrocytes with demyelination and relative astrocyte preservation. Multiple Sclerosis illustrates unilateral optic neuritis, periventricular “Dawson’s fingers” lesions, and multiple short-segment (<2 segments) peripheral spinal cord lesions, driven by T-cell/B-cell infiltration with oligoclonal bands. CTD-associated neuroinflammation shows variable optic nerve involvement and spinal cord lesions with vascular features, mediated by small-vessel vasculitis and perivascular lymphocytic infiltration. The bottom comparison highlights the key distinguishing feature: central cord (NMOSD), conus-involving LETM (MOGAD), peripheral/short-segment (MS), and variable with vascular features (CTD). Abbreviations: AQP4-IgG, aquaporin-4 immunoglobulin G; MOG-IgG, myelin oligodendrocyte glycoprotein immunoglobulin G; LETM, longitudinally extensive transverse myelitis; CTD, connective tissue disease.

Comparison with NMOSD and MS: The key distinguishing features of MOGAD versus NMOSD and MS are summarized in Table 1 and Figure 2. MOGAD occupies an intermediate position: it shares the LETM pattern with NMOSD but the demyelinating pathology and favorable steroid response with MS. Recognition of MOGAD as a distinct entity has critical therapeutic implications, as therapies effective for MS (interferon-*β*, fingolimod, natalizumab) may be ineffective or exacerbate disease, while the targeted NMOSD biologics lack approval for MOGAD. Accurate differentiation among these three conditions is therefore essential for appropriate treatment selection and optimal patient outcomes.

**Table 1:** Differential diagnosis of neuroimmunological overlap syndromes.

Feature	NMOSD (AQP4-IgG+)	MOGAD	MS	SLE (NPSLE)	SS	RA (CNS)
Primary target	Astrocytes (AQP4)	Myelin (MOG)	Myelin/ oligodendrocytes	Endothelium/neurons	Small vessels	Vasculature/ cervical spine
Core clinical features	ON + LETM, APS, intractable hiccups/nausea	ON, myelitis, ADEM-like	ON, myelitis, brainstem, cerebellar	Cognitive, seizures, psychosis, ON, TM	Sensory ataxia, ON, TM, small-fiber neuropathy	Cervical myelopathy, peripheral neuropathy
Optic neuritis pattern	Bilateral, severe, posterior > anterior, poor recovery	Often bilateral, severe, good recovery with steroids	Unilateral, mild–moderate, good recovery	Unilateral or bilateral, variable	Unilateral, variable	Rare
Spinal cord MRI	LETM (≥3 segments), central cord, T2-hyperintense, swelling	LETM (often long), conus involvement	Short segment (<2), peripheral, <1/2 cord cross-section	LETM possible, often with SLE features	LETM possible	Cervical cord compression (rheumatoid pannus)
Brain MRI	Periependymal, hypothalamus, brainstem, normal early	Often normal, deep white matter, thalamus	Dawson’s fingers, juxtacortical, infratentorial, corpus callosum	Non-specific white matter hyperintensities, atrophy	Non-specific white matter lesions	Non-specific white matter changes
CSF findings	Neutrophilic pleocytosis, elevated protein, rare OCBs	Lymphocytic pleocytosis, elevated protein, rare OCBs	Lymphocytic pleocytosis, OCBs+ (90%), elevated IgG index	Mild pleocytosis, elevated protein, rare OCBs	Mild pleocytosis, elevated protein	Normal or mild changes
Key autoantibodies	AQP4-IgG (95% specificity)	MOG-IgG (transient)	None specific	ANA, anti-dsDNA, anti-Smith, aPL, anti-Ro/SSA	Anti-Ro/SSA, anti-La/SSB, ANA	RF, anti-CCP
Therapy response	B-cell depletion +++, Complement inhibition +++, IL-6 blockade +++	Steroids +++, IVIG +++, B-cell depletion ±	DMTs (interferon, anti-CD20, S1P modulators)	Steroids, cyclophosphamide, rituximab, belimumab	Steroids, rituximab, IVIG	DMARDs, anti-TNF, rituximab, tocilizumab
Therapies to avoid	Interferon-β, fingolimod, natalizumab	None specific	None specific	None specific	None specific	None specific
Prognosis	Relapsing, severe disability if untreated	Monophasic or relapsing, better recovery	Relapsing–remitting → secondary progressive	Variable, depends on organ involvement	Usually, benign neurological course	Progressive if untreated

Therapeutic response categories reflect the strength of supporting evidence: “+++” indicates support from randomized controlled trials; “++” indicates support from prospective cohort or observational studies; “+” indicates support from case series or expert consensus. Therapies to avoid are based on case reports and observational evidence of disease exacerbation.

### 4.3 Multiple sclerosis

Multiple sclerosis is a chronic autoimmune demyelinating disease of the CNS characterized by inflammatory plaques, axonal degeneration, and progressive neurological dysfunction. Unlike NMOSD, MS primarily targets myelin and oligodendrocytes rather than astrocytes. The disease is thought to result from complex interactions between genetic susceptibility, environmental factors, and dysregulated immune responses involving autoreactive T cells and B cells. Optic neuritis is one of the most common initial manifestations of MS and often presents with unilateral painful visual loss and impaired color perception (74). Compared with NMOSD, visual recovery in MS-associated optic neuritis is generally more favorable. Myelitis in MS is usually partial and shorter in length than the LETM lesions observed in NMOSD (52).

MRI is essential for MS diagnosis and monitoring. Characteristic findings include multiple ovoid hyperintense lesions in the periventricular, juxtacortical, infratentorial, and spinal cord regions on T2-weighted imaging. The classic “Dawson’s fingers” appearance reflects lesions oriented perpendicular to the ventricles. Spinal cord lesions in MS are typically shorter than two vertebral segments and involve peripheral cord regions rather than the central cord distribution commonly seen in NMOSD (75). Cerebrospinal fluid (CSF) analysis often reveals oligoclonal bands and elevated IgG index in MS patients, findings less common in NMOSD. Importantly, some therapies effective for MS, such as interferon-beta and fingolimod, may exacerbate NMOSD, highlighting the necessity for accurate differential diagnosis (76).

### 4.4 Clinical manifestations and MRI findings

The clinical manifestations of autoimmune optic neuritis and myelitis vary depending on the underlying disease mechanism and anatomical localization of inflammation. Common symptoms include visual impairment, eye pain, limb weakness, numbness, gait instability, sensory deficits, neuropathic pain, and autonomic dysfunction. Severe cases may lead to blindness, paralysis, respiratory failure, or permanent disability. MRI remains the cornerstone imaging modality for evaluating CNS inflammatory disorders. In optic neuritis, orbital MRI with gadolinium enhancement can identify optic nerve inflammation, swelling, and enhancement patterns. NMOSD-associated optic neuritis involves long optic nerve segments and posterior visual pathways, whereas MS-associated lesions are typically shorter and unilateral. Spinal MRI findings also aid differential diagnosis. LETM lesions extending over three or more vertebral segments strongly suggest NMOSD or autoimmune myelitis associated with CTDs. In contrast, MS lesions are usually shorter, patchy, and asymmetrical.

Brain MRI can demonstrate disease-specific patterns including periventricular lesions in MS and hypothalamic or periependymal lesions in NMOSD. Early recognition of these radiological and clinical differences is crucial because prompt immunotherapy significantly improves neurological outcomes and reduces long-term disability. As neuroimmunological research advances, integration of imaging biomarkers, autoantibody profiling, and molecular diagnostics will continue to refine disease classification and therapeutic strategies (77). The comparative clinical and radiological features of optic neuritis and myelitis across NMOSD, MOGAD, MS, and CTD-associated neuroinflammation are illustrated in Figure 2. While optic neuritis and myelitis represent the clinical and radiological manifestations of central nervous system (CNS) inflammation, the underlying systemic autoimmunity often originates from connective tissue disorders (CTDs). The following section examines how systemic lupus erythematosus, Sjögren syndrome, and rheumatoid arthritis-each with distinct autoantibody profiles and pathogenic mechanisms contribute to CNS injury and expand the neuroimmunological overlap spectrum.

## 5 Connective tissue disorders and neurological involvement

Connective tissue disorders (CTDs) are systemic autoimmune diseases characterized by chronic immune activation, multi-organ involvement, and production of a broad spectrum of autoantibodies. Neurological manifestations in CTDs are increasingly recognized as part of a neuroimmunological continuum that overlaps with disorders such as neuromyelitis optica spectrum disorder (NMOSD), multiple sclerosis (MS)-like presentations, optic neuritis, and myelitis. These neurological complications arise from a combination of systemic inflammation, vasculopathy, autoantibody-mediated injury, and breakdown of immune privilege within the central nervous system (CNS). Among CTDs, systemic lupus erythematosus (SLE), Sjögren syndrome (SS), and rheumatoid arthritis (RA) represent the most frequently associated conditions with CNS involvement in the context of neuroimmunological overlap syndromes (78).

### 5.1 Systemic lupus erythematosus (SLE)

Systemic lupus erythematosus is a prototypic systemic autoimmune disease with highly heterogeneous clinical manifestations, including significant neurological involvement collectively termed neuropsychiatric SLE (NPSLE) (79). CNS complications in SLE may include optic neuritis, transverse myelitis, seizures, cognitive dysfunction, psychosis, and cerebrovascular disease. The pathogenesis of neurological involvement in SLE is multifactorial. Autoantibody-mediated injury plays a central role, particularly involving anti-dsDNA and anti-phospholipid antibodies, which contribute to endothelial dysfunction, microthrombosis, and blood–brain barrier (BBB) disruption (80). BBB impairment allows peripheral immune mediators and autoreactive lymphocytes to access CNS tissue, triggering local inflammatory cascades and neuronal injury. Myelitis in SLE may clinically and radiologically resemble NMOSD, particularly in cases associated with anti-AQP4 antibodies or coexisting autoimmune overlap (39). Notably, most cases of longitudinally extensive transverse myelitis (LETM) in SLE are likely associated with AQP4 antibodies and reflect an overlap of NMOSD and SLE rather than a pure manifestation of SLE. Optic neuritis can also occur as an isolated or concurrent manifestation, often with variable visual recovery depending on inflammatory severity and vascular involvement. Importantly, the overlap between SLE-associated myelitis and NMOSD highlights shared immunopathogenic pathways, including complement activation, cytokine dysregulation, and astrocytic injury (81).

### 5.2 Sjögren syndrome (SS)

Sjögren Syndrome is a chronic autoimmune disorder primarily affecting exocrine glands but increasingly recognized as a systemic disease with significant neurological involvement. CNS manifestations include optic neuritis, myelitis, sensory ataxia, cognitive dysfunction, and demyelinating-like syndromes (82).

Neurological complications in SS are thought to arise from small-vessel vasculitis, lymphocytic infiltration, and immune-mediated neuronal damage. In some patients, SS is associated with anti-AQP4 antibodies, linking it directly to NMOSD and reinforcing its role as a key CTD in neuroimmunological overlap syndromes (83). The same is likely true for LETM in SS, which frequently coincides with AQP4-IgG seropositivity and represents an overlap between SS and NMOSD rather than isolated SS-related myelitis. Myelitis in SS presents as longitudinally extensive spinal cord lesions, closely mimicking NMOSD both clinically and radiologically. Peripheral nervous system involvement is also common in SS, including sensory neuropathies and autonomic dysfunction (55). These manifestations further support the concept of widespread neuroimmune dysregulation affecting both central and peripheral compartments.

### 5.3 Rheumatoid arthritis (RA)

Rheumatoid Arthritis is a chronic systemic inflammatory disease primarily affecting synovial joints but also associated with extra-articular neurological complications (84). Although CNS involvement is less common compared to SLE and SS, RA has been increasingly linked to neuroinflammatory and neurovascular abnormalities. Neurological manifestations in RA include peripheral neuropathy, cervical myelopathy due to atlantoaxial subluxation, and rare but significant CNS complications such as vasculitis, demyelination, and cerebral ischemia (85). Chronic systemic inflammation in RA contributes to endothelial dysfunction and BBB disruption, facilitating immune cell trafficking into the CNS. Pro-inflammatory cytokines such as TNF-*α*, IL-6, and IL-1β play central roles in both joint destruction and neuroinflammation (86, 87). These mediators may promote microglial activation and neuronal sensitization, potentially contributing to cognitive dysfunction, mood disorders, and increased risk of depression and anxiety in RA patients.

In addition, long-term corticosteroid uses and immunomodulatory therapies may further influence CNS susceptibility to vascular and inflammatory injury. RA patients are known to have a higher chance of developing psychiatric comorbidities including depression and anxiety, mood, and psychotic disturbances, which greatly reduce quality of life and physical and mental health (88). Similarly, compared with patients without physical disability or recurring pain, mental health disorders in RA patients are associated with harmful effects such as fatigue, impaired sleep quality, increased mental health-related distress, and passive pain-coping strategies (89). Psychiatric comorbidities also adversely affect multiple outcomes in RA patients, including poor medication adherence and treatment response. For example, RA patients with depression are associated with an inferior response to biological therapy (90).

### 5.4 Other autoimmune disorders

Beyond classical CTDs, several other autoimmune conditions are increasingly associated with optic neuritis, myelitis, and broader neuroimmunological overlap syndromes. Autoimmune thyroid disease, systemic vasculitides, and mixed connective tissue disease (MCTD) have all been reported in association with CNS demyelination and inflammatory optic neuropathies ((52, 91)). Rare but severe neuroimmunological complications have also been described, including cases of autoimmune encephalitis followed by hemophagocytic lymphohistiocytosis, a life-threatening hyperinflammatory syndrome that requires prompt recognition and aggressive management (92).

**Figure 3:**
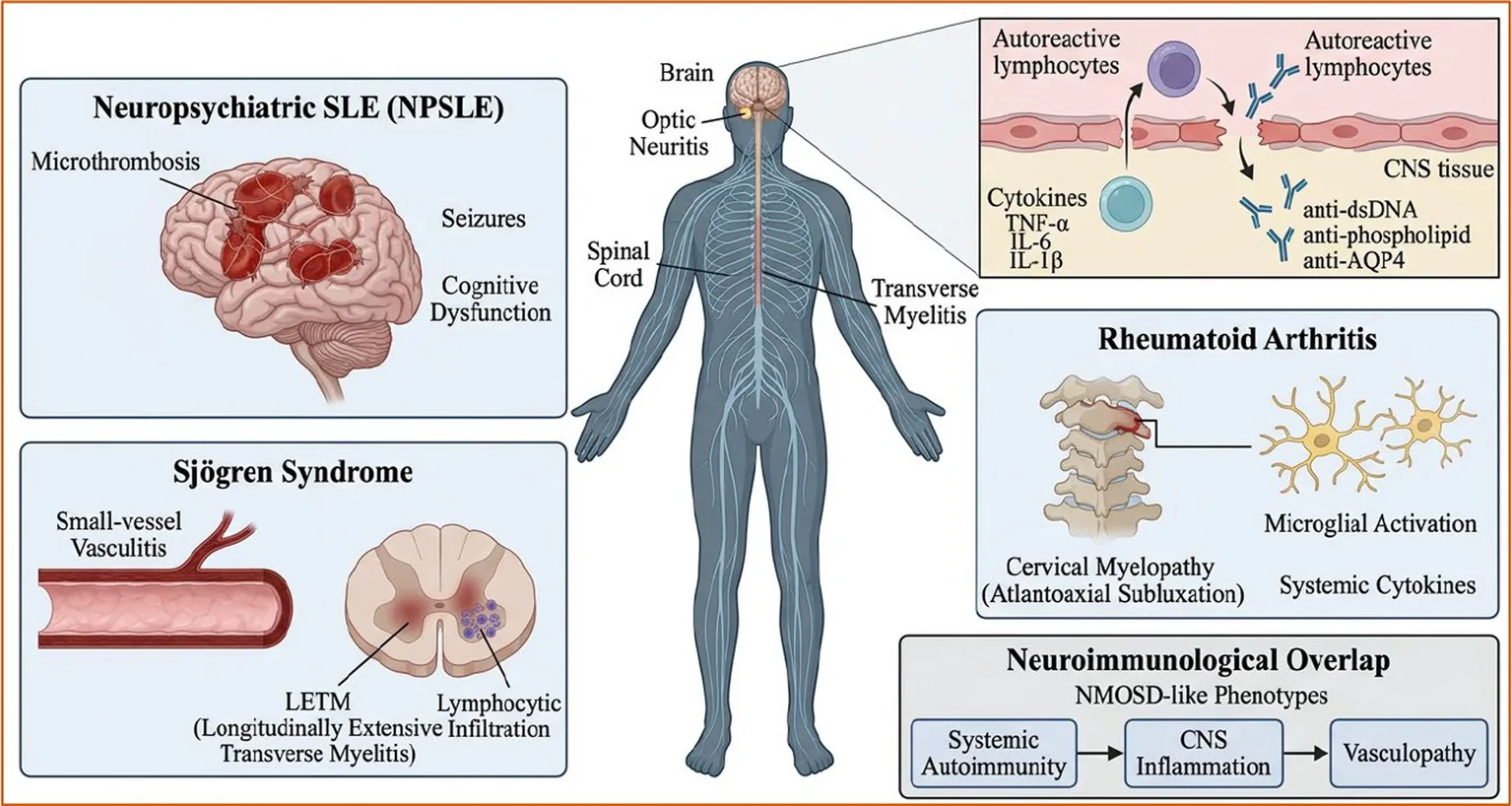
Neurological involvement in connective tissue disorders (CTDs). Overview of central nervous system (CNS) manifestations associated with major connective tissue disorders. Upper-left box, neuropsychiatric systemic lupus erythematosus (NPSLE): cerebral microthrombosis, seizures, and cognitive dysfunction are illustrated on the brain schematic; pathogenic mechanisms include anti-dsDNA, antiphospholipid, and anti-AQP4 autoantibodies acting on autoreactive lymphocytes that cross the BBB under the influence of TNF-*α*, IL-6, and IL-1*β* (central inset, upper right). Lower-left box, Sjögren syndrome: small-vessel vasculitis and a spinal cord cross-section depict longitudinally extensive transverse myelitis (LETM) with perivascular lymphocytic infiltration. Lower-right box, rheumatoid arthritis: cervical myelopathy secondary to atlantoaxial subluxation is shown alongside microglial activation driven by systemic cytokines. The central body schematic indicates the principal anatomical sites of involvement (brain, optic nerve, spinal cord) shared across these conditions. The bottom diagram summarizes the common downstream pathway by which systemic autoimmunity and CNS inflammation converge on vasculopathy to produce NMOSD-like phenotypes across CTDs.

In systemic vasculitis, immune complex deposition and vascular inflammation can lead to ischemic injury of the optic nerve and spinal cord. Mixed connective tissue disease, characterized by overlapping features of SLE, SS, and systemic sclerosis, may also present with demyelinating or NMOSD-like phenotypes, particularly in patients with overlapping autoantibody profiles. Collectively, these disorders underscore the spectrum nature of neuroimmunological disease, in which systemic autoimmunity and CNS inflammation converge through shared molecular pathways. The presence of overlapping clinical phenotypes and autoantibody signatures reinforces the need for comprehensive immunological and neuroimaging evaluation in patients presenting with optic neuritis or myelitis in the context of systemic autoimmune disease. The spectrum of central nervous system manifestations associated with systemic lupus erythematosus, Sjögren syndrome, and rheumatoid arthritis, along with shared pathogenic pathways, is summarized in Figure 3.

## 6 Diagnosis and biomarkers

**Figure 4:**
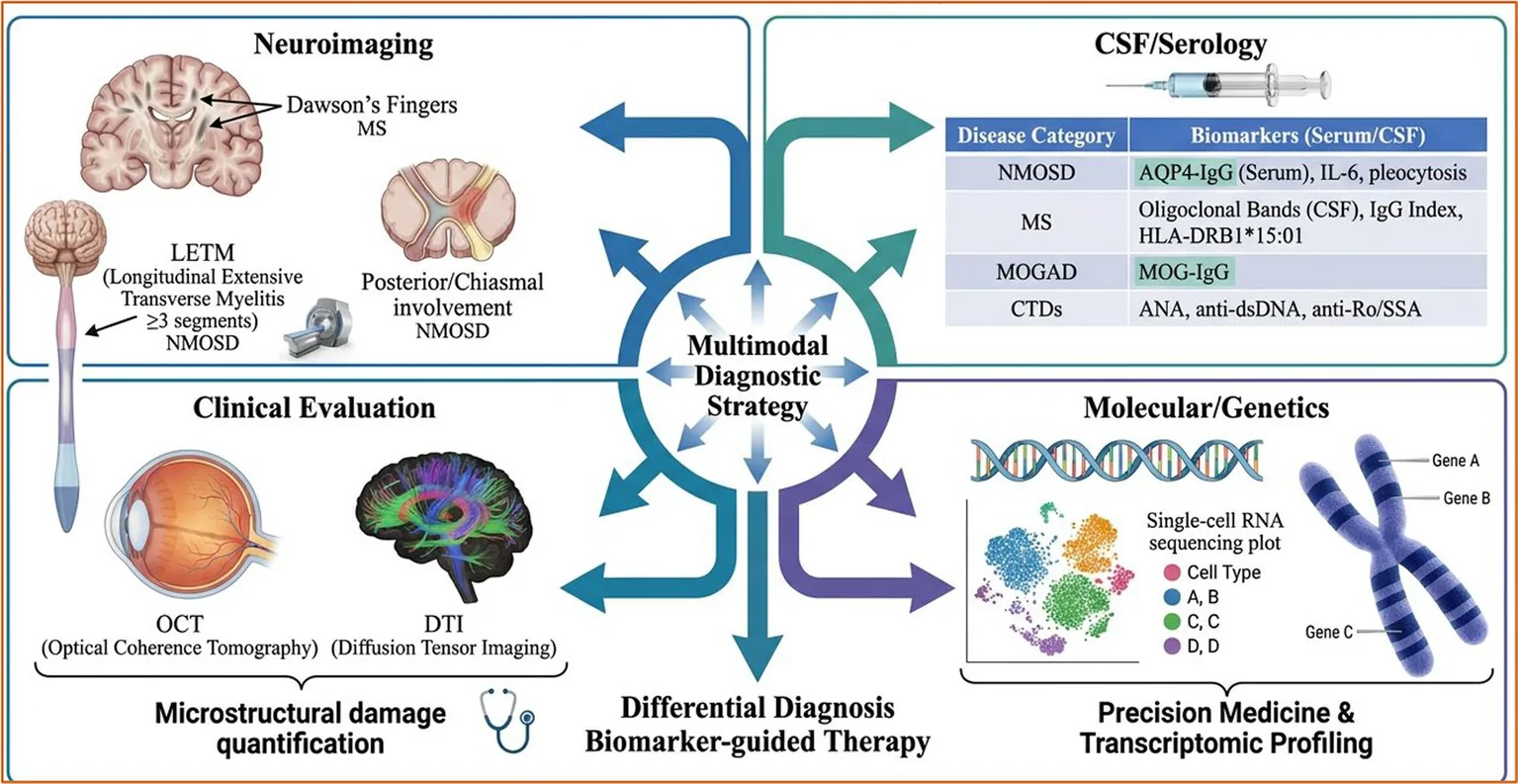
Integrated diagnostic approach and biomarkers for neuroimmunological overlap syndrome. Multimodal diagnostic algorithm for evaluating patients with suspected neuroimmunological overlap syndromes presenting with optic neuritis and/or myelitis, organized around a central “Multimodal Diagnostic Strategy” hub that integrates four complementary quadrants. Upper-left quadrant, Neuroimaging: periventricular “Dawson’s fingers” on brain MRI (characteristic of MS) versus longitudinally extensive transverse myelitis (LETM, ≥3 vertebral segments) and posterior/chiasmal optic nerve involvement on spinal and orbital MRI (characteristic of NMOSD). Upper-right quadrant, CSF/Serology: a disease-category table linking NMOSD to AQP4-IgG (serum), IL-6, and CSF pleocytosis; MS to oligoclonal bands, IgG index, and HLA-DRB1*15:01; MOGAD to MOG-IgG; and CTDs to ANA and anti-Ro/SSA. Lower-left quadrant, Clinical Evaluation: optical coherence tomography (OCT) and diffusion tensor imaging (DTI) for microstructural damage quantification. Lower-right quadrant, Molecular/Genetics: single-cell RNA sequencing and genetic/chromosomal analysis supporting precision medicine and transcriptomic profiling. Integration of these four quadrants enables accurate differentiation among NMOSD, MOGAD, MS, and CTD-associated neuroinflammation, guiding biomarker-informed differential diagnosis and targeted therapy.

The diagnosis of neuroimmunological overlap syndromes involving optic neuritis, myelitis, and connective tissue disorders (CTDs) relies on an integrated approach combining clinical evaluation, neuroimaging, cerebrospinal fluid (CSF) analysis, serological testing, and emerging molecular and genetic biomarkers. Given the phenotypic overlap between disorders such as NMOSD, MS, SLE, SS, and RA, accurate diagnosis is often challenging and requires careful interpretation of multimodal data. Early and precise identification is essential to guide appropriate immunotherapy and prevent irreversible neurological damage (40, 41). Table 1 summarizes the key differentiating features among NMOSD, MOGAD, MS, and CTD-associated neurological involvement, including primary targets, clinical patterns, MRI characteristics, CSF findings, autoantibodies, and therapeutic responses. An integrated diagnostic algorithm combining clinical assessment, neuroimaging, cerebrospinal fluid analysis, and serological biomarkers for accurate differentiation of neuroimmunological overlap syndromes is presented in Figure 4.

### 6.1 Neuroimaging

Neuroimaging, particularly magnetic resonance imaging, is a cornerstone in the diagnostic workup of neuroimmunological overlap syndromes. MRI enables the visualization of inflammatory lesions, demyelination, and structural damage within the optic nerves, spinal cord, and brain.

In optic neuritis, orbital MRI with gadolinium contrast is used to detect optic nerve inflammation, swelling, and enhancement (93). In neuromyelitis optica spectrum disorder (NMOSD), lesions typically involve long segments of the optic nerve, often extending posteriorly toward the optic chiasm, and are associated with severe visual impairment and poor recovery (94). In contrast, optic neuritis in Multiple Sclerosis (MS) usually demonstrates shorter, unilateral lesions with more favorable visual outcomes.

Spinal cord MRI is particularly valuable for distinguishing NMOSD from MS and CTD-associated myelitis. NMOSD is characterized by longitudinally extensive transverse myelitis (LETM), defined as spinal cord lesions spanning three or more vertebral segments, often centrally located with cord swelling (94, 95). In contrast, MS-related myelitis typically presents with short-segment, peripheral lesions. In CTDs such as systemic lupus erythematosus and Sjögren syndrome, spinal lesions may mimic NMOSD but are often accompanied by systemic vascular or inflammatory features (40, 41).

Brain MRI findings further aid differentiation. NMOSD commonly affects periependymal regions rich in aquaporin-4 expression, including the hypothalamus, brainstem, and periventricular areas. MS, on the other ha, is characterized by ovoid periventricular lesions (“Dawson’s fingers”), juxtacortical plaques, and infratentorial involvement (96). In CTD-associated neuroinflammation, imaging findings are often nonspecific and may reflect vasculitic, ischemic, or inflammatory processes.

Advanced imaging modalities, including diffusion tensor imaging (DTI), magnetization transfer imaging, and optical coherence tomography (OCT), are increasingly used to detect microstructural damage and quantify axonal loss, particularly in optic neuritis and chronic myelitis.

### 6.2 CSF and serological biomarkers

Cerebrospinal fluid analysis yields essential diagnostic and prognostic data in neuroimmunological overlap syndromes. Standard CSF parameters include cell count, protein concentration, immunoglobulin synthesis, and oligoclonal band (OCB) detection (97).

In MS, the presence of CSF-restricted OCBs and elevated IgG index is a characteristic finding. In contrast, OCBs are less frequently detected in NMOSD, and CSF may show neutrophilic or mixed pleocytosis during acute attacks. Elevated CSF protein and leukocyte counts are also observed in severe inflammatory episodes, particularly in NMOSD and CTD-associated myelitis (98).

Serological biomarkers play a central role in distinguishing between overlapping neuroimmunological conditions. The most clinically significant antibody is aquaporin-4 immunoglobulin G (AQP4-IgG), which is highly specific for NMOSD and directly pathogenic through complement-mediated astrocyte injury (99). The detection of AQP4-IgG in serum is considered diagnostic in the appropriate clinical context (100). AQP4-IgG demonstrates approximately 95% specificity for NMOSD and is detectable in approximately 70–80% of patients with the full clinical syndrome (101).

Another important biomarker is myelin oligodendrocyte glycoprotein antibody (MOG-IgG), associated with MOG antibody-associated disease (MOGAD), which presents with optic neuritis, myelitis, and acute disseminated encephalomyelitis-like syndromes (47). MOGAD is now recognized as a separate disease entity from both NMOSD and MS, supported by converging serological, pathological, and clinical evidence, and is associated with distinct treatment responses that have direct implications for management (28). In contrast to AQP4-IgG-seropositive NMOSD, MOGAD more frequently follows a monophasic course, and patients typically show better recovery from acute attacks and a more favorable response to corticosteroids, with some patients not requiring long-term maintenance immunotherapy after a single event (102). When relapses do occur, optic neuritis predominates over myelitis, in contrast to the more symmetric distribution seen in NMOSD. Critically, the approved NMOSD biologics discussed in Section 6.2-eculizumab, ravulizumab, satralizumab, and inebilizumab-were developed and validated specifically in AQP4-IgG-seropositive cohorts and are not approved for MOGAD; rituximab and maintenance oral immunosuppression (azathioprine, mycophenolate) remain the principal options for relapsing MOGAD, reflecting the current absence of MOGAD-specific pivotal trial data.

In CTDs, a wide range of autoantibodies contribute to diagnosis and disease stratification. These include antinuclear antibodies (ANA), anti-double-stranded DNA (anti-dsDNA), anti-Ro/SSA, anti-La/SSB, and antiphospholipid antibodies (29). Their presence is particularly relevant in neuropsychiatric SLE and Sjögren-related neurological disease, where they may correlate with vasculopathy, BBB disruption, and CNS inflammation.

Inflammatory cytokines such as IL-6 and IL-17 are emerging CSF biomarkers in NMOSD, reflecting active disease and B-cell-mediated pathology. Elevated complement activation products have also been associated with disease severity and tissue injury in antibody-mediated demyelination (40, 41).

Beyond disease-defining autoantibodies, glial fibrillary acidic protein (GFAP) and neurofilament light chain (NfL) have emerged as promising research for monitoring astrocytic and axonal injury, respectively. However, it is critical to distinguish their current status from that of established diagnostic biomarkers. Serum GFAP is markedly elevated during NMOSD attacks and tends to normalize with effective complement- or B-cell-targeted therapy, making it a candidate biomarker of disease activity (103). In contrast to GFAP, which is relatively specific to astrocytic injury, NfL is a more generic marker of axonal damage that rises in NMOSD, MS, and MOGAD relapses alike, and has shown prognostic value for residual disability (77, 104). However, neither biomarker has yet been prospectively validated for routine clinical decision-making, and neither has replaced AQP4-IgG or MOG-IgG serology as a primary diagnostic test. The combination of GFAP and NfL may help distinguish NMOSD from MS and MOGAD when antibody testing is equivocal, with GFAP elevation favoring NMOSD and isolated NfL elevation suggesting MS or MOGAD (105–107). However, neither biomarker has yet replaced AQP4-IgG or MOG-IgG serology as a primary diagnostic test, and their role in routine clinical practice continues to be defined through prospective studies.

Single-cell immune profiling and high-dimensional cytometry have enabled unprecedented characterization of pathogenic immune cell subsets involved in neuroimmunological overlap syndromes. Single-cell RNA sequencing has identified clonally expanded B-cell populations in both CSF and peripheral blood of NMOSD patients, revealing plasmablast signatures that correlate with disease activity (106, 108). These studies have demonstrated that plasmablasts are the primary source of AQP4-IgG production and are enriched during active disease. Similarly, single-cell analysis has uncovered distinct Th17 and regulatory T-cell dysfunction profiles in MS and CTD-associated neuroinflammation, providing insights into disease-specific immunopathology (31, 109). In SLE patients with neuropsychiatric manifestations, single-cell profiling has identified type I interferon signatures in CSF immune cells, linking systemic interferon activation to CNS inflammation (19).

Proteomic profiling of CSF and serum has identified disease-specific protein signatures that differentiate NMOSD from MS and MOGAD. Elevated complement components (C3a, C5a, sC5b-9), inflammatory cytokines (IL-6, IL-17, TNF-*α*), and chemokines (CXCL13, CCL2, CXCL10) have been associated with disease activity and relapse risk (20). In NMOSD, the complement activation signature is particularly prominent, reflecting the pathogenesis of AQP4-IgG-mediated injury. In MS, proteomic profiling has revealed a predominant T-cell and microglial activation signature, while CTD-associated neuroinflammation shows an interferon-inducible protein signature. The combination of proteomic biomarkers with clinical and imaging data is being explored to develop predictive models for treatment response and disease progression, with machine learning approaches showing promise in integrating these multi-dimensional datasets.

Transcriptomic analysis of peripheral blood mononuclear cells has revealed distinct gene expression profiles in NMOSD, MS, and CTD-associated neurological disease. NMOSD is characterized by enhanced humoral immunity and B-cell activation signatures, including upregulation of genes involved in antibody production, plasma cell differentiation, and complement activation (12, 110). MS demonstrates a predominant T-cell-mediated inflammatory profile with activation of Th1 and Th17 pathways, while CTD-associated neuroinflammation shows interferon and innate immune pathway activation, reflecting the systemic interferon signature characteristic of SLE and SS. These molecular signatures are being integrated into multi-omics frameworks to refine disease classification, predict treatment responses, and identify novel therapeutic targets.

### 6.3 Molecular and genetic approaches

Advances in molecular biology and genetics have significantly improved understanding of susceptibility and pathogenesis in neuroimmunological overlap syndromes. Genetic predisposition, particularly within the human leukocyte antigen (HLA) system, plays a key role in modulating immune responses and disease risk.

In MS, associations with HLA-DRB1*01:01 are well established, whereas NMOSD shows weaker and more heterogeneous HLA associations, suggesting distinct immunogenetic mechanisms. In CTDs such as SLE and SS, multiple susceptibility loci have been identified, including genes involved in interferon signaling, B-cell activation, and immune regulation (111–113).

Transcriptomic and proteomic profiling have revealed disease-specific molecular signatures that reflect underlying immune dysregulation. For example, NMOSD is characterized by enhanced humoral immunity and plasmablast expansion, whereas MS demonstrates a stronger T-cell-mediated inflammatory profile. These differences support the concept of distinct but overlapping immunopathological pathways (114).

Emerging molecular techniques, including single-cell RNA sequencing and high-dimensional immune profiling, have enabled detailed characterization of immune cell subsets involved in CNS inflammation. These approaches have identified pathogenic Th17 cells, dysfunctional regulatory T cells, and clonally expanded B-cell populations in both CNS and peripheral compartments (105).

Genetic and molecular biomarkers are increasingly being integrated with clinical and imaging data to develop precision medicine approaches. Such multimodal strategies aim to improve diagnostic accuracy, predict disease course, and guide targeted therapies in patients with overlapping neuroimmunological and systemic autoimmune disorders. Accurate diagnosis through integrated neuroimaging, CSF analysis, and serological biomarkers provides the essential foundation for therapeutic decision-making. The following section translates these diagnostic insights into clinical action, reviewing current and emerging treatment strategies-from conventional immunosuppression to targeted biologics tailored to the underlying immunopathology of each disease subtype.

## 7 Therapeutic strategies

**Figure 5:**
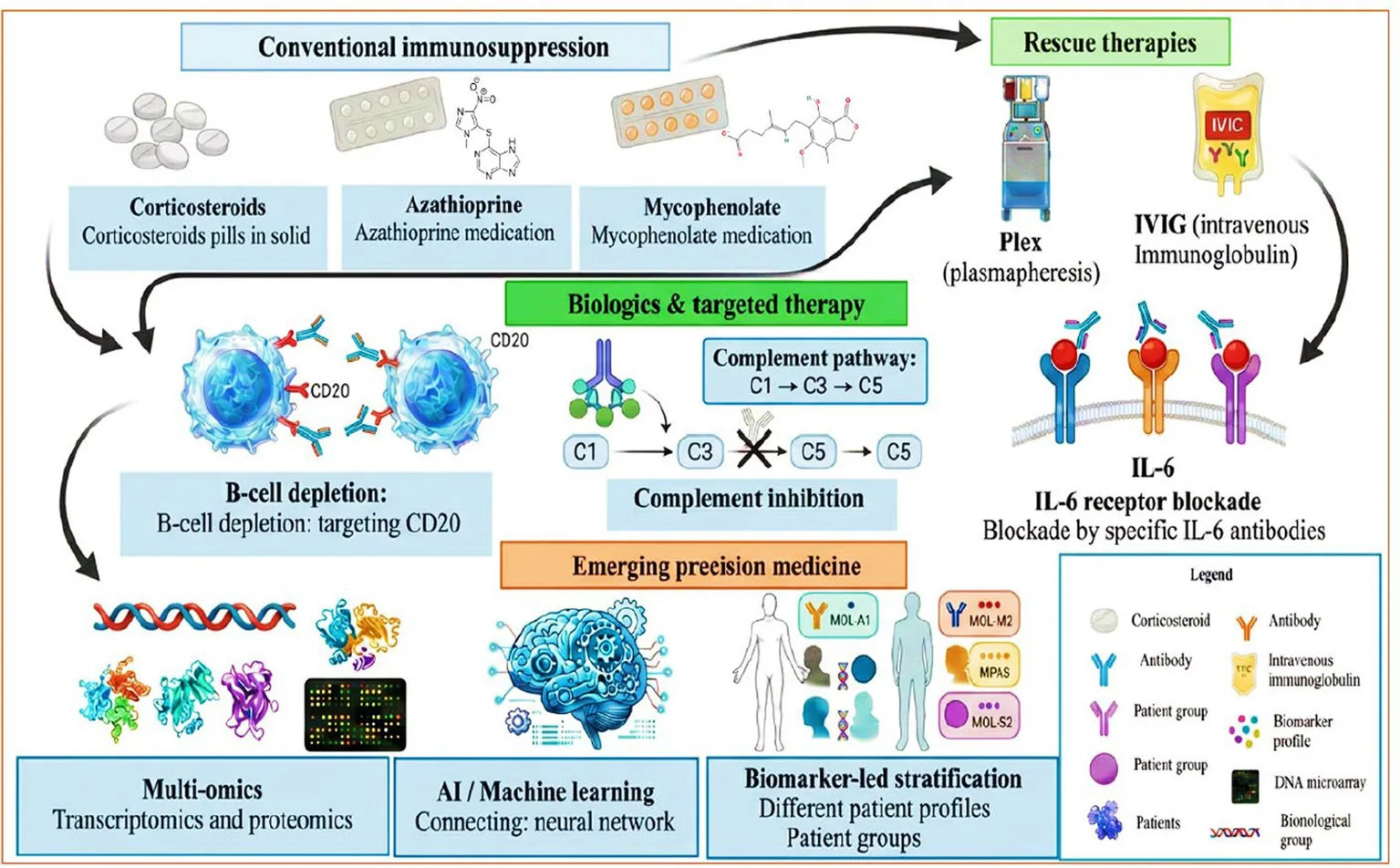
Therapeutic strategies and emerging precision medicine approaches. Schematic illustration of current and emerging therapeutic approaches. Approved therapies (eculizumab, ravulizumab, satralizumab, inebilizumab for AQP4-IgG-positive NMOSD) are shown in green; investigational or off-label approaches (rituximab, azathioprine, mycophenolate for MOGAD) are shown in orange; experimental/research-stage approaches (multi-omics integration, AI models) are shown in blue. The evolution from broad immunosuppression to targeted biologics and precision-guided therapy represents the paradigm shift in management.

Managing neuroimmunological overlap syndromes demands a tailored, multidisciplinary approach. Therapeutic goals encompass suppressing acute inflammation, preventing relapses, reducing long-term disability, and controlling underlying systemic autoimmunity. Because these conditions span a spectrum from T-cell-mediated to antibody-driven pathology, therapeutic selection must be guided by disease subtype, biomarker status, and clinical severity (Figure 5).

### 7.1 Immunosuppressive therapies

Conventional immunosuppressive agents remain the cornerstone of long-term management for many neuroimmunological overlap syndromes, particularly in disorders such as systemic lupus erythematosus (SLE), Sjögren syndrome (SS), and systemic manifestations of rheumatoid arthritis (RA) (115). These therapies aim to broadly reduce immune activation and prevent ongoing tissue damage.

High-dose corticosteroids are typically first-line treatment for acute inflammatory attacks, including optic neuritis and transverse myelitis (64, 116). Intravenous methylprednisolone is commonly used to rapidly suppress CNS inflammation and reduce edema. However, long-term steroid use is limited by adverse effects such as osteoporosis, metabolic syndrome, and increased infection risk.

Steroid-sparing immunosuppressants such as azathioprine, mycophenolate mofetil, methotrexate, and cyclophosphamide are widely used for maintenance therapy (117). In CTD-associated neuroinflammation, cyclophosphamide is often reserved for severe or refractory disease, particularly in lupus-related CNS involvement or vasculitic complications.

Plasma exchange (PLEX) and intravenous immunoglobulin (IVIG) are important rescue therapies in severe or steroid-refractory cases. PLEX is particularly effective in antibody-mediated conditions such as NMOSD, where removal of pathogenic autoantibodies can significantly improve neurological outcomes (118, 119). Recent real-world evidence from a multicenter study in China demonstrated that lymphoplasmapheresis an innovative technique integrating plasma exchange with lymphopheresis significantly reduced Expanded Disability Status Scale (EDSS) scores, decreased AQP4 antibody levels, and lowered proinflammatory cytokines in steroid-refractory NMOSD patients (120).

### 7.2 Biologics and targeted therapy

The development of biologic therapies has transformed the treatment landscape of antibody-mediated neuroimmunological diseases, particularly neuromyelitis optica spectrum disorder (NMOSD), which is strongly associated with aquaporin-4 immunoglobulin G (AQP4-IgG).

B-cell depletion therapies targeting CD20-positive cells, such as rituximab, have demonstrated substantial efficacy in reducing relapse rates in NMOSD, MS-like overlap syndromes, and CTD-associated CNS disease (36). Four biologic agents are now specifically approved for AQP4-IgG-seropositive NMOSD and illustrate distinct mechanistic approaches to B-cell- and cytokine-targeted therapy. Inebilizumab, an afucosylated anti-CD19 monoclonal antibody, depletes a broader B-lineage repertoire than anti-CD20 agents-including CD19-positive plasmablasts and some plasma cells not targeted by CD20-directed therapies-and reduced the risk of NMOSD attack relative to placebo in the pivotal N-MOmentum trial, a randomized trial conducted predominantly in AQP4-IgG-seropositive participants (121). Satralizumab, a recycling anti-IL-6 receptor monoclonal antibody, reduced relapse risk in both AQP4-IgG-seropositive and -seronegative NMOSD in the SAkuraSky and SAkuraStar trials, although the magnitude of benefit was substantially greater in the seropositive subgroup (122).

Eculizumab and the longer-acting ravulizumab, both terminal complement (C5) inhibitors, prevent assembly of the membrane attack complex and produced a large reduction in relapse risk relative to placebo in the PREVENT and CHAMPION-NMOSD trials, respectively; both trials enrolled exclusively AQP4-IgG-seropositive participants, so this magnitude of benefit should not be assumed to generalize to seronegative NMOSD or MOGAD (71, 123). Rituximab and ocrelizumab remain anti-CD20 options used largely off-label or as second-line therapy, supported mainly by observational rather than placebo-controlled trial data. Because these agents differ mechanistically-B-lineage depletion (inebilizumab, rituximab, ocrelizumab) versus cytokine receptor blockade (satralizumab) versus complement blockade (eculizumab, ravulizumab)-treatment selection in clinical practice is increasingly individualized according to comorbidity profile, dosing interval, and seropositivity status.

It is essential to recognize that the strength of evidence supporting therapeutic recommendations varies substantially across disease subtypes. For AQP4-IgG-seropositive NMOSD, four biologic agents-inebilizumab, satralizumab, eculizumab, and ravulizumab are supported by pivotal randomized controlled trials and have received regulatory approval. For MOGAD, by contrast, no targeted biologic has been approved, and treatment recommendations rely primarily on observational studies, case series, and expert consensus. Corticosteroids and intravenous immunoglobulin remain the mainstay of acute and maintenance therapy, respectively, reflecting the current absence of MOGAD-specific trial data. For CTD-associated neuroinflammation, evidence is even more heterogeneous, with most recommendations extrapolated from studies of the underlying systemic disease or from NMOSD literature. Clinicians should therefore exercise caution in generalizing treatment approaches across diagnostic boundaries and should base therapeutic decisions on the specific disease subtype, biomarker status, and available evidence quality.

Complement inhibition has emerged as a highly effective strategy in AQP4-IgG-positive NMOSD. Complement-mediated astrocytic injury is a key pathogenic mechanism, and drugs targeting this pathway have shown strong clinical benefit by preventing downstream inflammatory cascades (124). In addition to B-cell and complement-targeted therapies, interleukin pathway inhibition is an evolving area of treatment. IL-6 receptor blockade is particularly relevant in NMOSD, where IL-6 contributes to B-cell survival, plasmablast differentiation, and BBB disruption (125, 126). This therapeutic approach directly addresses the cytokine-driven amplification loop that underlies disease activity.

For CTD-associated neurological involvement, biologics targeting tumor necrosis factor-alpha (TNF-*α*), B cells, and T-cell co-stimulation pathways are also used, particularly in refractory RA or SLE with CNS manifestations. However, careful selection is required, as some biologics may exacerbate demyelinating disease in susceptible patients.

### 7.3 Emerging precision medicine approaches

The future of treatment for neuroimmunological overlap syndromes is increasingly focused on precision medicine, which integrates clinical phenotyping, serological biomarkers, neuroimaging, and molecular profiling to guide individualized therapy.

A key component of this approach is stratification based on autoantibody status. For example, patients with AQP4-IgG-positive NMOSD respond best to B-cell depletion, complement inhibition, or IL-6 blockade, whereas MOG antibody-associated disease (MOGAD) may require different immunosuppressive strategies and often shows better response to corticosteroids and IVIG (65).

Multi-omics approaches, including transcriptomics, proteomics, and metabolomics, are being explored to identify disease-specific molecular signatures that can predict treatment response and relapse risk. These techniques enable more refined classification of patients beyond traditional clinical diagnostic categories.

Artificial intelligence (AI) and machine learning are increasingly being applied to integrate large-scale datasets from imaging, laboratory biomarkers, and electronic health records. These tools may improve early diagnosis, predict disease trajectories, and optimize treatment selection in complex overlap syndromes. Another promising direction is immune cell-targeted therapy based on single-cell profiling, which allows selective modulation of pathogenic T-cell and B-cell subsets while preserving protective immunity. This could reduce the risk of generalized immunosuppression and associated complications.

Overall, the evolution from broad immunosuppression to targeted biologic therapy and precision-guided intervention represents a major paradigm shift in the management of neuroimmunological overlap syndromes, with the potential to significantly improve neurological outcomes and quality of life. While targeted biologic therapies have substantially improved outcomes in antibody-mediated neuroimmunological diseases, significant challenges remain in early diagnosis, disease stratification, and prevention of irreversible neurological disability. The following section looks forward to emerging innovations including neuroimmune therapeutics, personalized medicine, and multi-omics approaches integrated with artificial intelligence that promise to address these unmet needs.

## 8 Future perspectives

Neuroimmunological overlap syndromes involving optic neuritis, myelitis, and connective tissue disorders (CTDs) represent a rapidly evolving field at the intersection of neurology, immunology, and systems biology. Despite significant advances in understanding pathophysiology and improving targeted therapies, major challenges remain in early diagnosis, disease stratification, and prevention of irreversible neurological damage. Future progress is expected to be driven by advances in neuroimmune therapeutics, personalized medicine, and integrative multi-omics and artificial intelligence (AI)-based approaches.

### 8.1 Neuroimmune therapeutics

Future therapeutic strategies are likely to move beyond generalized immunosuppression toward precise modulation of neuroimmune interactions. A growing understanding of bidirectional communication between the nervous and immune systems suggests that targeting neuroimmune circuits may represent a novel therapeutic frontier.

Preclinical studies have identified a neural circuit in which corticotropin-releasing hormone neurons in the paraventricular nucleus (PVN) regulate splenic immune cells, modulating antibody responses. Following splenic nerve removal after vaccination, the number of antibody-secreting cells produced by mice decreased sharply, indicating that impulse signals of the splenic nerve promote humoral adaptive immune responses (127, 128). These findings imply that a central-peripheral nerve circuit directly regulates lymphocyte-mediated adaptive immune responses, which could represent the biological basis for immune response behavioral modulation. Such neuroimmune axes, including vagal and splenic signaling pathways, offer new opportunities to control autoimmune activation at its source rather than solely suppressing downstream inflammation (1, 129). Interventions targeting autonomic nervous system pathways or neuropeptide signaling could potentially reduce aberrant immune activation in diseases such as rheumatoid arthritis and systemic lupus erythematosus. Understanding the interactions between immunity, endocrinology, and the nervous system opens avenues for novel drugs aiming to modulate inflammatory pathological processes and improve prognosis.

In parallel, next-generation biologics are being developed to selectively inhibit key inflammatory mediators such as IL-6, complement components, and B-cell survival factors (130). These therapies are expected to become more refined, with improved safety profiles and reduced systemic immunosuppression. Cell-based therapies, including engineered regulatory T cells and B-cell modulation strategies, are being investigated as potential disease-modifying interventions. Chimeric antigen receptor (CAR) T-cell therapy, which has shown remarkable efficacy in hematological malignancies, is now being explored for autoimmune diseases, with preclinical studies demonstrating the potential of CAR-Tregs to suppress pathogenic autoreactive B cells (131). Similarly, B-cell tolerizing therapies that selectively induce apoptosis of autoreactive B cells while preserving protective humoral immunity are under development (132).

### 8.2 Personalized medicine

Personalized medicine is expected to play a central role in transforming the management of neuroimmunological overlap syndromes. Given the heterogeneity of diseases such as neuromyelitis optica spectrum disorder (NMOSD), multiple sclerosis (MS), and CTD-associated neuroinflammation, individualized treatment strategies based on molecular and clinical profiling are increasingly necessary (37, 110, 133).

Stratification based on autoantibody status (e.g., AQP4-IgG, MOG-IgG, ANA, anti-dsDNA) already represents an early form of precision medicine (134). Future approaches will expand this concept by integrating immune phenotyping, cytokine profiling, and genetic susceptibility markers to define patient-specific disease endotypes rather than broad diagnostic categories.

This shift is particularly important in overlap syndromes, where patients may present with mixed features of CNS demyelination and systemic autoimmunity. Personalized treatment algorithms will enable clinicians to select therapies that target dominant pathogenic pathways, such as B-cell driven versus T-cell driven inflammation, thereby improving efficacy and reducing unnecessary immunosuppression.

### 8.3 Multi-omics and AI approaches

Multi-omics technologies and artificial intelligence (AI) are poised to transform research and clinical practice in neuroimmunological diseases. Genomics, transcriptomics, proteomics, metabolomics, and epigenomics offer comprehensive molecular insights into disease mechanisms and immune dysregulation.

In diseases like systemic lupus erythematosus and NMOSD, multi-omics profiling has already begun to reveal distinct immune signatures associated with disease activity, treatment response, and relapse risk. Single-cell sequencing technologies further enable characterization of rare pathogenic immune cell subsets, including autoreactive B cells and pro-inflammatory T-cell populations involved in CNS injury (108).

Artificial intelligence and machine learning algorithms can integrate these complex datasets with clinical, imaging, and serological data to identify predictive patterns that are not discernible through traditional analytical methods. AI-based models have the potential to improve early diagnosis, predict disease progression, and optimize therapeutic selection in real time.

In neuroimaging, AI-assisted analysis of MRI and optical coherence tomography (OCT) data may enhance detection of subtle structural changes in optic nerves and spinal cord lesions (135–137). Similarly, predictive modeling may help identify patients at high risk of developing severe neurological complications, enabling earlier intervention.

It is important to acknowledge that while these approaches are promising, they remain largely at the research stage. Multi-omics profiling, single-cell sequencing, and AI-based decision support tools have not yet been prospectively validated for routine clinical decision-making in neuroimmunological overlap syndromes (40, 41). The evidence base for these technologies currently derives from single-center cohorts, retrospective analyses, and proof-of-concept studies, rather than large-scale prospective multicenter trials. Distinguishing established diagnostic biomarkers (AQP4-IgG, MOG-IgG, CSF oligoclonal bands) from emerging but unvalidated candidates (GFAP, NfL, transcriptomic signatures) is essential for appropriate clinical interpretation. Similarly, AI models require rigorous external validation across diverse patient populations before they can be integrated into clinical practice. The current state of the field is therefore one of rapid exploration and discovery, with cautious optimism for translation, rather than established clinical deployment.

### 8.4 Limitations and unresolved controversies

Several limitations and unresolved questions temper the optimism of the preceding sections and merit explicit acknowledgment.

First, this is a narrative rather than systematic review; sources were selected for thematic relevance rather than through a pre-registered, reproducible search strategy, and the literature base is necessarily skewed toward NMOSD, for which biomarker and trial data are most mature, relative to MOGAD and CTD-associated neuroinflammation, for which the evidence is sparser and more heterogeneous.

Second, much of the mechanistic synthesis presented here-particularly shared cytokine networks and complement pathways across NMOSD, MS, SLE, SS, and RA-is extrapolated from disease-specific studies rather than head-to-head comparative cohorts, and the degree to which these pathways are truly convergent versus superficially similar remains incompletely tested.

Third, emerging biomarkers such as GFAP and NfL, and precision-medicine tools such as single-cell transcriptomics and AI-based decision support, are presented as promising research directions; none has yet been prospectively validated for routine clinical decision-making in this overlap population, and their inclusion here should be read as a research agenda rather than current standard of care.

Fourth, treatment recommendations drawn from pivotal NMOSD trials (e.g., for satralizumab, eculizumab, ravulizumab, and inebilizumab) apply specifically to AQP4-IgG-seropositive patients and should not be generalized to MOGAD, seronegative NMOSD, or CTD-associated neuroinflammation, for which dedicated trial data are largely lacking.

Finally, whether neuroimmunological overlap syndromes truly constitute a unified “neuroimmune continuum,” as proposed in the abstract and conclusion, or instead represent mechanistically distinct diseases that merely share downstream clinical phenotypes, remains an open and actively debated question that future longitudinal and multi-omics studies will need to resolve.

## 9 Conclusion

Neuroimmunological overlap syndromes encompassing optic neuritis, myelitis, and connective tissue disorders represent a paradigm of interconnected systemic and neurological autoimmunity. This review has synthesized current evidence demonstrating that these conditions share convergent molecular mechanisms blood–brain barrier dysfunction, cytokine dysregulation, complement activation, and adaptive immune imbalance that transcend traditional diagnostic boundaries. Viewing these conditions as a neuroimmune continuum, rather than discrete diseases, provides a more accurate representation of their biological reality and explains why central nervous system inflammation and systemic autoimmunity so frequently co-occur.

The clinical implications of this framework are substantial. In diagnostic practice, evaluation should move beyond categorical assignment to comprehensively assess the relative contribution of astrocytopathy, demyelination, microglial activation, and vasculopathy in each patient. Accurate differentiation among NMOSD, MOGAD, MS, and CTD-associated neuroinflammation is essential because inappropriate immunotherapies can worsen outcomes. For instance, interferon beta and fingolimod, effective in MS, may exacerbate NMOSD, while the targeted biologics approved for AQP4-IgG-positive NMOSD are not validated for MOGAD. The recognition of MOGAD as a distinct entity with unique clinical, radiological, and therapeutic features further emphasizes the need for comprehensive diagnostic evaluation incorporating autoantibody profiling, neuroimaging, and cerebrospinal fluid analysis.

The therapeutic implications are equally significant. The shift from broad immunosuppression toward targeted biologics-B-cell depletion, complement inhibition, and IL-6 receptor blockade represents a paradigm change that aligns with the continuum model by targeting specific pathogenic pathways rather than disease labels. Four agents are now approved for AQP4-IgG-positive NMOSD, each addressing a distinct mechanism: inebilizumab (B-lineage depletion), satralizumab (IL-6 receptor blockade), and eculizumab and ravulizumab (C5 complement inhibition). The availability of these mechanistically diverse options enables individualized treatment selection based on patient-specific factors such as comorbidity profile, seropositivity status, and predicted response. However, the absence of approved targeted therapies for MOGAD and limited trial data for CTD-associated neuroinflammation highlight critical gaps that must be addressed through dedicated research.

Several priority areas for future research emerge from this analysis. First, prospective multicenter studies are urgently needed to validate emerging biomarkers such as GFAP and NfL for routine clinical use, as these currently remain promising but unproven candidates. Second, head-to-head comparative cohorts across NMOSD, MOGAD, MS, and CTD-associated neuroinflammation are required to determine the degree of molecular convergence versus divergence, directly testing the predictions of the continuum framework. Third, randomized controlled trials specifically targeting MOGAD and CTD-associated neuroinflammation are critically needed to establish evidence-based treatment recommendations. Fourth, longitudinal multi-omics studies with extended follow-up are essential to map the dynamic evolution of molecular signatures over time and identify predictors of disease course and treatment response.

The central unresolved question is whether neuroimmunological overlap syndromes truly constitute a unified continuum or instead represent mechanistically distinct diseases that merely share downstream clinical phenotypes. This question can only be resolved through prospective studies integrating longitudinal clinical, imaging, and multi-omics data. Additionally, the optimal biomarkers for monitoring disease activity, the mechanisms underlying the transition from systemic autoimmunity to central nervous system inflammation, and the identification of patients at highest risk for severe neurological complications remain incompletely understood.

In summary, neuroimmunological overlap syndromes require reconceptualization from discrete diagnostic categories toward an integrated framework that acknowledges shared pathogenic mechanisms while respecting disease-specific differences. Progress in understanding neuroimmune crosstalk, combined with advances in biomarker discovery and targeted therapy, has already begun to reshape clinical practice. Continued integration of translational research, advanced imaging, and computational biology will be essential to fully unravel disease complexity. The remaining challenges including the lack of approved therapies for MOGAD, the need for validated biomarkers, and the unresolved question of the continuum hypothesis represent priority areas for future research that will determine whether this framework becomes an established clinical paradigm or remains a heuristic for organizing current knowledge. Ultimately, this convergence of neuroimmunology, precision medicine, and data-driven approaches holds promise for earlier diagnosis, more effective individualized treatment, and improved long-term outcomes for patients affected by these challenging disorders.
